# Molecular and Microscopic Analysis of Bacteria and Viruses in Exhaled Breath Collected Using a Simple Impaction and Condensing Method

**DOI:** 10.1371/journal.pone.0041137

**Published:** 2012-07-25

**Authors:** Zhenqiang Xu, Fangxia Shen, Xiaoguang Li, Yan Wu, Qi Chen, Xu Jie, Maosheng Yao

**Affiliations:** 1 State Key Joint Laboratory of Environmental Simulation and Pollution Control, College of Environmental Sciences and Engineering, Peking University, Beijing, China; 2 Department of Infectious Disease, Peking University Third Hospital, Peking University, Beijing, China; University of Hong Kong, Hong Kong

## Abstract

Exhaled breath condensate (EBC) is increasingly being used as a non-invasive method for disease diagnosis and environmental exposure assessment. By using hydrophobic surface, ice, and droplet scavenging, a simple impaction and condensing based collection method is reported here. Human subjects were recruited to exhale toward the device for 1, 2, 3, and 4 min. The exhaled breath quickly formed into tiny droplets on the hydrophobic surface, which were subsequently scavenged into a 10 µL rolling deionized water droplet. The collected EBC was further analyzed using culturing, DNA stain, Scanning Electron Microscope (SEM), polymerase chain reaction (PCR) and colorimetry (VITEK 2) for bacteria and viruses.

Experimental data revealed that bacteria and viruses in EBC can be rapidly collected using the method developed here, with an observed efficiency of 100 µL EBC within 1 min. Culturing, DNA stain, SEM, and qPCR methods all detected high bacterial concentrations up to 7000 CFU/m^3^ in exhaled breath, including both viable and dead cells of various types. *Sphingomonas* paucimobilis and *Kocuria* variants were found dominant in EBC samples using VITEK 2 system. SEM images revealed that most bacteria in exhaled breath are detected in the size range of 0.5–1.0 µm, which is able to enable them to remain airborne for a longer time, thus presenting a risk for airborne transmission of potential diseases. Using qPCR, influenza A H3N2 viruses were also detected in one EBC sample. Different from other devices restricted solely to condensation, the developed method can be easily achieved both by impaction and condensation in a laboratory and could impact current practice of EBC collection. Nonetheless, the reported work is a proof-of-concept demonstration, and its performance in non-invasive disease diagnosis such as bacterimia and virus infections needs to be further validated including effects of its influencing matrix.

## Introduction

Bioaerosols are present virtually anywhere in the environment, and their exposure is shown to cause numerous adverse health effects [1]–[2]. In addition, there is also a possible release of bio-warfare agents in a man-made bio-terror event. A number of studies demonstrated that the respiratory tract can be colonized with disease organisms [3]–[5]. Through talking, coughing, sneezing or singing, the potential virulent organisms can be exhaled and spread into the ambient environment [6], which accordingly causes air contamination. For example, SARS in 2003 and H1N1 in 2009 outbreaks were shown to be attributed to the airborne route of disease transmission [7]–[10].

Among many other diseases, respiratory infection accounts for 23.3–42.1% of the total hospital infections [11], and is listed as the third leading killer [12]. However, present diagnosis procedures using nasal swabs, bronchoalveolar lavages, nasopharyngeal aspirates or sputum samples, appear to cause unpleasant experiences in addition to long detection time. During flu outbreaks, body temperature or isolation procedures are often used to control and prevent further spread, however such methods are lacking scientific evidence and not always effective with those patients infected but in latent period. On another front, exhaled breath condensate (EBC) as a simple and noninvasive method is increasingly being utilized in early disease screening and infectious aerosols measurements, e.g., lung cancer [13], [14], asthma [15], [16], and other respiratory problems [17], [18]. In previous studies, human influenza A viruses were detected in exhaled breath using EBC [19], [20] as well as filter [21], mask [22], [23] and a liquid sampler [24]. In another study, foot-and-mouth disease viruses were also found in the exhaled air from experimentally infected cattle [25]. In addition, high levels of bacterial concentrations in EBC were also observed in other studies [26]–[29]. It was recently shown that exhaled breath could be also analyzed for fungal infection by relevant biomarker, e.g., 2-Pentyl furan (2PF) for aspergillosis [30]. Overall, EBC has demonstrated great potential and advantages in early disease screening and diagnosis [31], opening a new arena for studying airway inflammation and chemistry [32]. Recently, Vereb et al (2011) suggested that exhaled breath can be also used for assessing a variety of environmental exposures [33].

For EBC related studies, the first key step is the collection of exhaled breath. Over the years, a variety of devices (Table S1, Supporting Information) were developed including Rtube collection system (Respiratory Research, Inc, Charlottesville, VA) and EcoScreen® condenser (Erich Jaeger Gmbh, Wurzbur, Germany). Typically, these devices would be able to collect 1000 µl of EBC samples within about 10 min, however the collection often comes with a lengthy procedure and a higher cost. For example, use of the EcoScreen involves 7 steps: 1) turn on to cool, 2) clean collection tube, 3) clean condensation chamber insert, 4) retrieve cooling sleeve from freezer, 5) sample collection, 6) sample storage and transport, 7) removal of sample (Respiratory Research, Inc, Charlottesville, VA). The RTube eliminates the first 3 steps, but each collection still requires 10 min and costs $23.25 (Respiratory Research, Inc, Charlottesville, VA) compared to 31 min and $47.17 per collection for the EcoScreen. These collection devices are generally expensive, e.g., the EcoScreen costs around $9000. A recent study compared the sampling efficiency of the Rtube (widely used EBC collection device) with that of throat swab method, showing detection rates of 7% and 46.8% for the Rtube and the throat swab method, respectively [20]. It was suggested that the RTube is not applicable for viral detection in exhaled breath [20]. In addition, condenser coatings [34], sampling temperature [35] and sampling times [36] were shown to affect physical collection efficiencies of available EBC collectors. Among others, the noted problems with these available EBC collection devices are the device availability, reusability and possible cross contamination [37], which would negatively impact their wide applications. In addition, EBC collection is strictly limited to the method condensation only in most studies [38]. To fully utilize EBC in early disease screening, diagnosis and environmental exposure assessment, simple yet efficient EBC collection device using different methods and biological characterization of the EBC sample are needed.

In this study, a novel EBC collection method was developed by using hydrophobic surface, a layer of ice, and a droplet scavenging procedure. The physical collection efficiency (amount of EBC collected per unit of time) of the device was evaluated. In addition, biological analysis and characterization of EBC samples collected from human subjects were conducted using culturing, DNA stain, SEM, qPCR and species identification tool VITEK 2. This work contributes to the effort in applying EBC together with molecular tools as a non-invasive method in rapid disease diagnosis.

## Materials and Methods

### Development of exhaled breath condensate collection device and method

**Figure 1:**
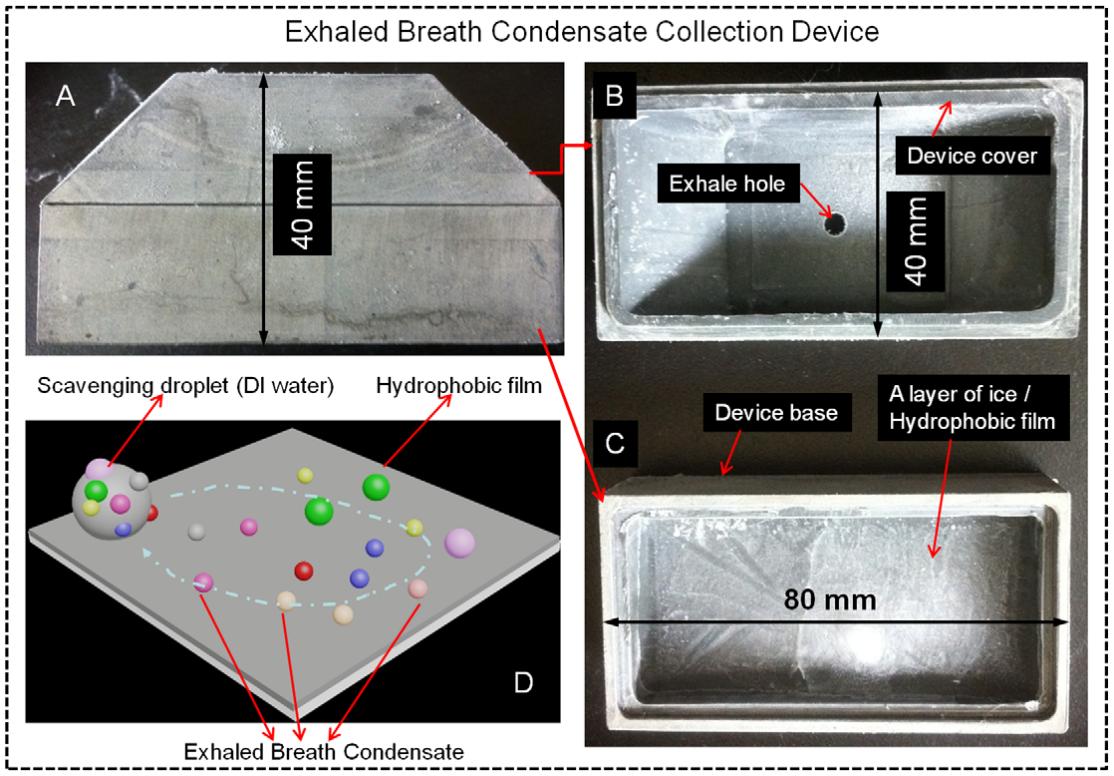
Exhaled breath condensate collection device (A) and method developed in this study: B) the EBC collection device cover, C) the collection device base with a layer of ice and hydrophobic film on the top, D) the exhaled breath condensate collection method: 5–10 µl DI water pipetted and scrolled over the hydrophobic film to scavenge EBC droplets.

**Figure 2:**
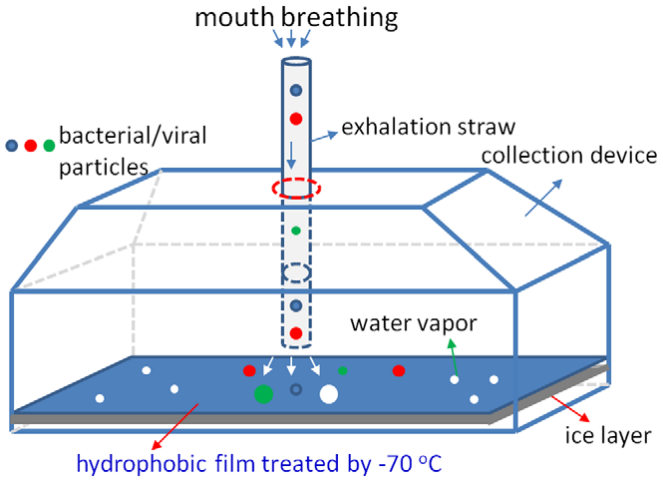
Sketch of experimental setup for collecting exhaled breath samples.

The collection method and device developed and experiential set up for collecting EBC are shown in Figure 1 and Figure 2, respectively. As observed in Figure 1, a simple EBC collection device was developed here. The EBC collection device is composed of four major parts as shown in Figure 1: collection device cover, collection device base, a layer of ice, and a hydrophobic film (treated by ultralow temperature −70°C). The collection device cover and base were made of Teflon™ polytetrafluoroethylene (PTFE) material, and a parafilm (Parafilm Co. Menasha, WI) used as the hydrophobic surface. The dimensions of the collection device are measured as 80×40×40 (mm) (length×width×height). In the collection device cover, there is a hole with a diameter of 6 mm as the exhaled breath inlet. The thickness of the collection device cover and base was about 3 mm, and the whole collection device weighs around 105 g. The layer of ice is used to keep the treated hydrophobic film cool.

For EBC collection, sterile water (DNA and RNA free) was first added into the collection base of the device up to a depth of 5 mm as observed in Figure 2. And then, the device base together with the cover was placed in an ultralow temperature (−70°C) refrigerator (Thermo Fisher Scientific Co. Marietta, OH) to form a layer of ice. Following this step, a sterile hydrophobic parafilm measured as 80×40×0.3 (mm)(length×width×thickness) was placed onto the surface of the ice suited in the collection base. To collect EBC samples, a disposable sterile straw with a diameter of 5 mm (16 cm long) is inserted through the exhaled breath inlet shown in Figure 2, with its end 2 mm above the hydrophobic parafilm. The human subjects are then advised to mouth-breathe without wearing a nose clip through the exhaled breath inlet shown in Figure 2 toward the hydrophobic film for a selected time (1–4 min). Due to the low temperature and hydrophobic nature of the parafilm surface, exhaled breath quickly condenses into tiny liquid droplets on the hydrophobic surface. Assuming an average breathing rate of 12 L/min for an adult, the particle speed from the exhaled breath would be around 10 m/s given the size of the straw (5 mm in diameter). Therefore, during the exhaled breath collection, the bacteria or virus particles would impact onto the hydrophobic surface at a speed of 10 m/s. In addition to condensing used for other EBC collection procedures, the method developed here also rely on the impaction to collect the bacterial and viral particles. Given such a speed, there might be possible particle bounce problems, however the bacterial or viral particles in the exhaled breath usually come with water droplets, which thus minimizes the potential particle bounce problem.

**Figure 3:**
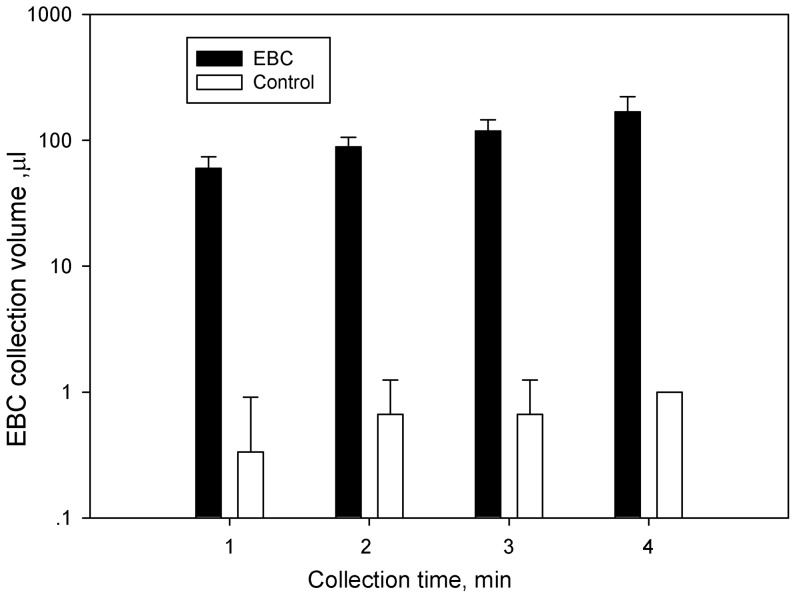
Physical collection efficiency of the exhaled breath condensate collection device developed in this study under different collection time; control indicates the total volume of sample collected without breathing toward the device; data points represent averages and standard deviations of six EBC collection volume data by different subjects.

**Figure 4:**
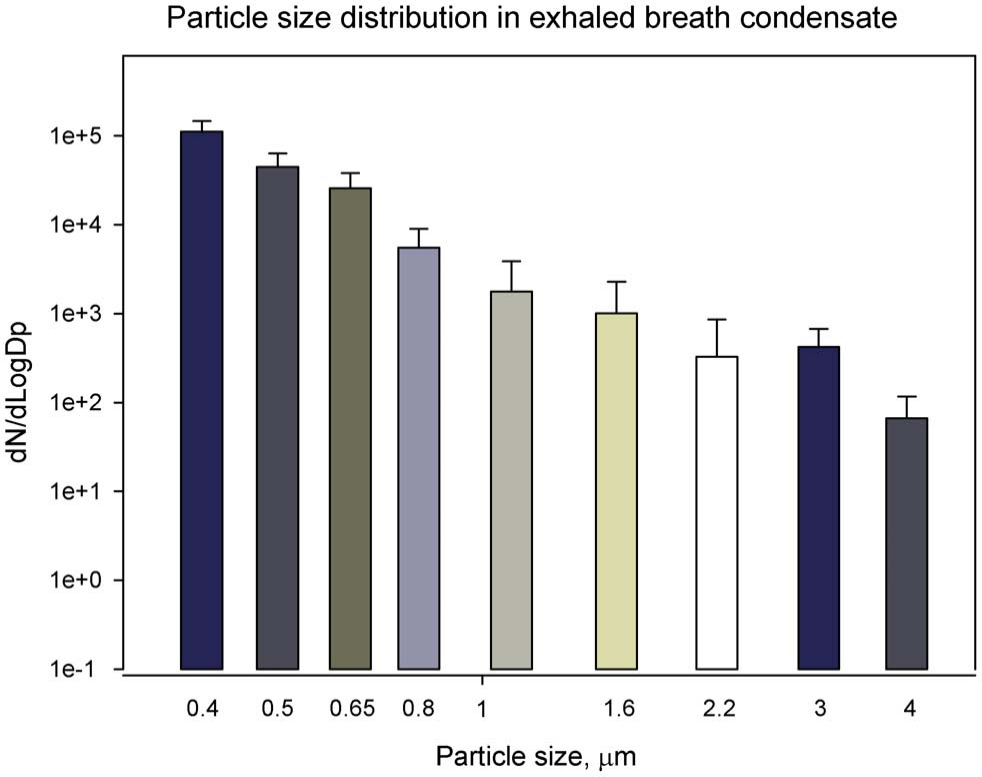
Particle size distributions in exhaled breath by mouth breathing using an Optical Particle Counter(OPC); x-axis shows the average diameters of 16 channel sizes of the OPC; data points represent averages and standard deviations of 20 min measurements by the OPC.

**Figure 5:**
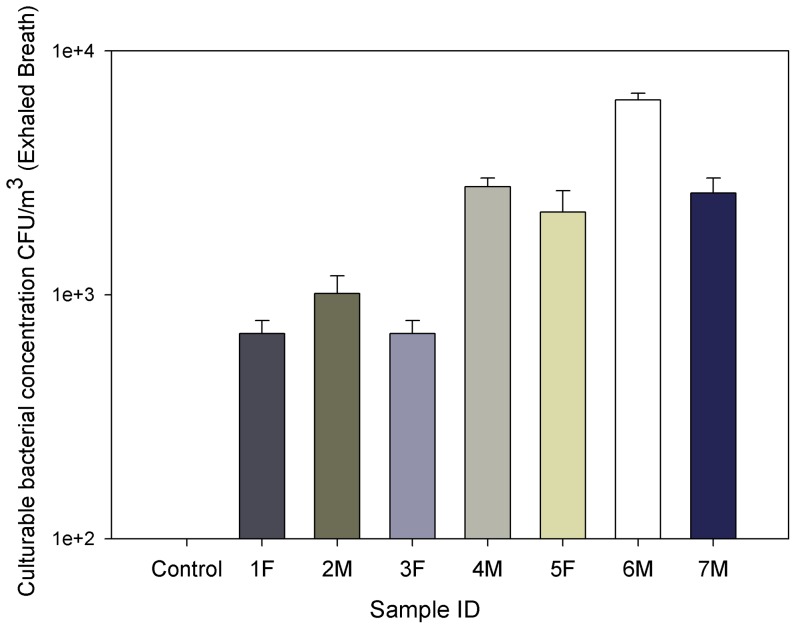
Culturable bacterial aerosol concentrations detected in exhaled breath condensate samples collected using the device from seven human subjects with symptoms listed in Table S2; F and M indicate Female and Male, respectively, 1–7 indicate the subject ID corresponding to those listed in Table S2; EBC collection time was 3 min; data points represent averages and standard deviations from at least three replicates.

After the collection, about 10 µl of DNA and RNA free DI water was pipetted onto the hydrophobic film as observed in Figure 1. To collect breath samples on the hydrophobic film, one only needs to use the pipette to touch the DI water droplet, and then drag the DI water droplet to scroll over the entire surface. The DI water droplet would move with the pipette without an extra step. The materials collected on the surface would be subsequently scavenged into the water droplet. After this operation, the collected EBC samples in the form of bigger liquid droplet as shown in Figure 1D were transferred to a sterile tube by a pipette for subsequent analysis. The samples collected without the exhaled breath from human subjects are used as the negative controls. The EBC collection efficiency and biological analysis of collected samples were performed as outlined in the experimental procedure shown in Figure S1 (Supporting Information).

### Amount and variability of EBC collected by the device developed

To investigate the amount of variability in EBC collected by the method developed, six student volunteers were recruited to exhale through the device for 1, 2, 3 and 4 min. The volume of collected EBC was measured by a calibrated pipette (Eppendorf, Hauppauge, NY). The amount of EBC per unit time collected by the device was determined using averages of EBC samples obtained by the volunteers under each of specific collection time tested. For each EBC collection, a different hydrophobic film and a different exhalation straw were used. In addition, the particle size distributions in the exhaled breath through mouth-breathing were also measured in a particle free bio-safety hood using an Optical Particle Counter (OPC) (GRIMM Co. Ltd., Ainring, Germany) at a flow rate of 1.2 L/min. To ensure air stream balance, the OPC was connected to a two-way tubing, which connects to clean air (Biological SafetyHood) and the breathing straw, respectively.

**Figure 6:**
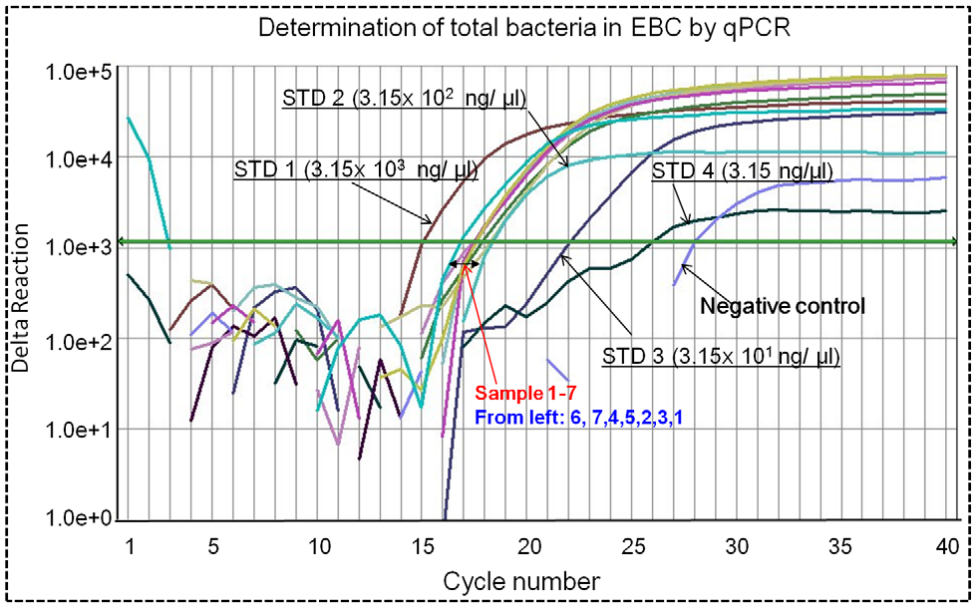
Determination of total bacterial aerosols in EBC by qPCR; DNA standards (STD) used were 3.15, 3.15×10^1^, 3.15×10^2^, 3.15×10^3^ ng/µl *Bacillus subtilis* DNA; Sample 1–7 represent EBC samples collected from seven human subjects with their medical conditions listed in Table S2; DI water was used as the negative control.

**Figure 7:**
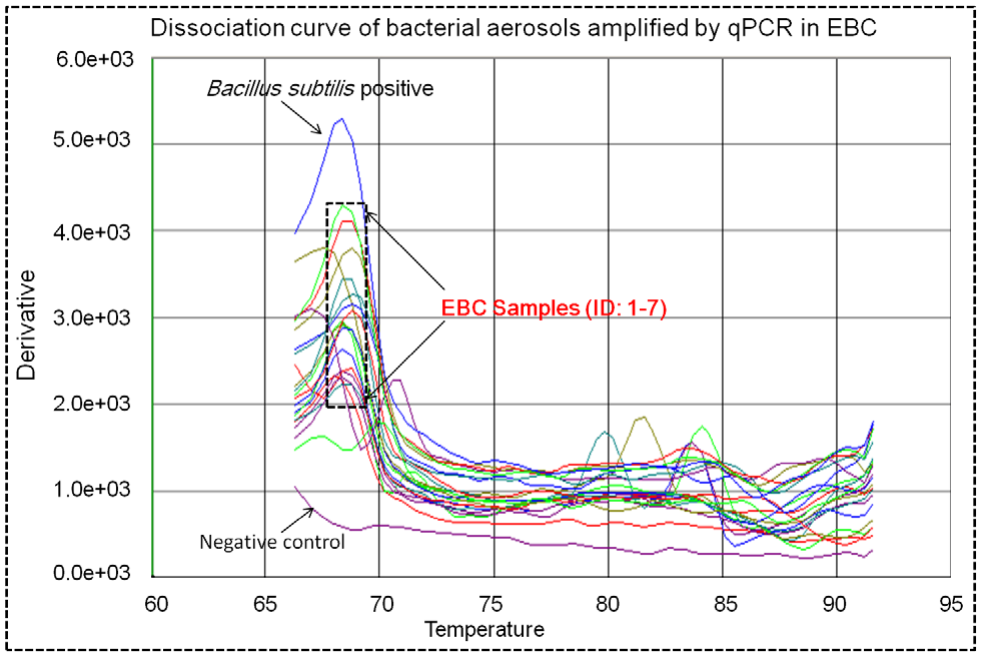
Dissociation curve of bacterial aerosols in EBC samples amplified by qPCR; Samples 1–7 were those collected from seven human subjects with their medical conditions listed in Table S2; *Bacillus subtilis* species was used as the positive control and DI water was used as the negative control; the curves shown here include two duplicates for each EBC sample.

**Figure 8:**
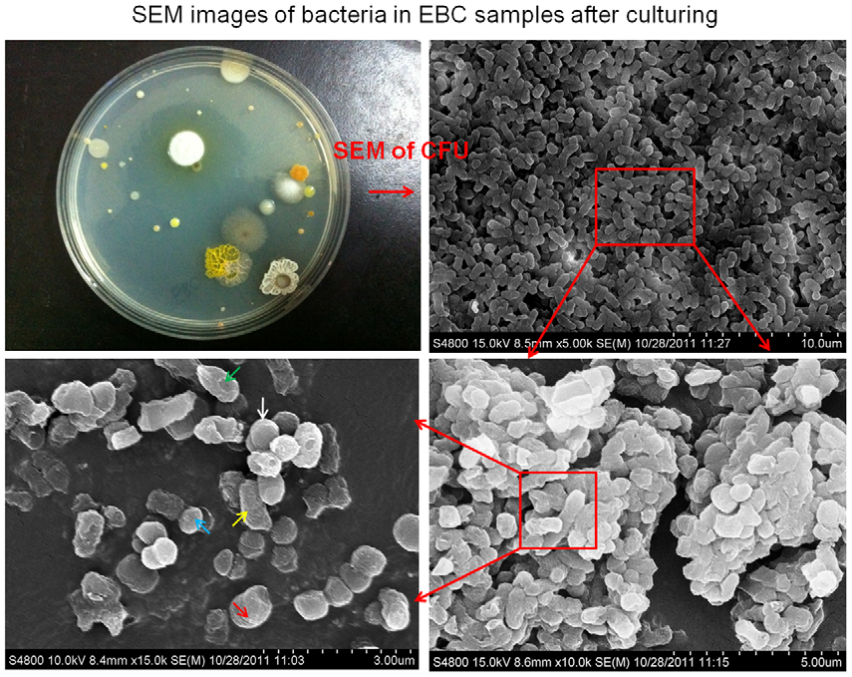
SEM images (different resolutions) of bacteria in EBC samples and images of colony forming units after culturing; the EBC samples were collected from human subjects and cultured using liquid broth overnight; different colored arrows point to likely different bacteria (different morphologies); *Sphingomonas* paucimobilis, *Kocuria* rosea, *Bacillus* lentus, *Aerococcus* viridians, *Bacillus* firmus, *Kocuria* kristinae, *Staph*. Xylosus were identified in EBC samples from patients with respiratory symptoms using VITEK 2 system.

### Bacterial and viral aerosol concentrations and species in EBC sample

In this work, seven patients with onset flu symptoms (their medical information is listed in Table S2, Supporting Information) were also recruited from the respiratory clinic of Peking University Third Hospital in Beijing. About 40 µl of exhaled breath condensate collected from each of 7 patients was diluted by 10 times and then plated on Trypticase Soy Agar (TSA) (Becton, Dickson and Company, Sparks, MD) plates at 26°C for 2–3 days, and colony forming units (CFUs) were manually counted. The total culturable bacterial aerosol concentration was calculated as CFU/m^3^ (exhaled breath) by considering the collection time and an average breathing rate of 12 L/min for an adult. Besides, the culturable bacterial species were identified using VITEK® 2 (BioMérieux, Inc,100 Rodolphe Street, Durham, NC). In addition, molecular detection of bacteria and virus using qPCR and RT-qPCR, respectively, were performed according to the procedures described in Supporting Information S1. To further confirm the bacterial presence DNA stain of EBC sample by Acridine Orange (AO) was also conducted.

### Statistical analysis

The differences in collected EBC volumes and culturable bacterial aerosol concentrations obtained by the EBC collection device were analyzed by Analysis of Variance (ANOVA). A p-value of less than 0.05 indicates a statistically significant difference at a confidence level of 95%. Collection of EBC from human subjects was approved by Peking University Ethnics Committee.

## Results and Discussion

Here, a novel EBC collection method and device was developed and evaluated in collecting EBC samples from human subjects using culturing and molecular methods. Compared to those currently available devices shown in Table S1, our device is lightweight with simplicity, reusability, and lower cost. The developed collection device itself costs less than $10, with about $0.5 for consumables (straw and hydrophobic film) per collection. The time needed for 100 µl EBC including sample collection and removal was around 2 min. The physical collection efficiency of the device is shown in Figure 3. The data points shown in the figure were averages of the EBC samples collected from six volunteers under each of the collection times (1, 2, 3 and 4 min) tested. In general, the amount of EBC sample collected was observed to increase with increasing collection time were observed among subjects. As also observed in Figure 3, the method has a good reproducibility (small variations). ANOVA analysis indicated that the collection time had a statistically significant effect on the amount of EBC sample collected per unit of time (p-value = 0.0026). For the 4 min collection, the volume of collected EBC (168.7 µL) was 1.8 times of that (60.0 µL in average) by 1 min. In our study, when no EBC was collected about 1 µL of liquid was obtained from the hydrophobic surface in an environment with a temperature of 17.9–19.3°C and a relative humidity level of 46–52%. In addition, during the breath sample collection, the collection device had a higher air pressure due to the exhaling, thus it is less likely that environmental air would come into the device. This suggests that environmental water vapor had limited impact on the collection method given the total amount of EBC collected. A recent study indicated that the minimum required volume of EBC was 50 µL for follow-up biological and chemical analysis, such as multiplexed cytokine analysis [35]. This on the other hand implies that the EBC device developed in this study can provide adequate amount of EBC sample for rapid analysis. Here, only one type of hydrophobic surface (parafilm) was tested, and in the future different hydrophobic materials should be also explored to improve the overall efficiency.

As listed in Table S1, currently available EBC collection devices, e.g., the Rtube and the EcoScreen, are comparable to ours with respect to rate of EBC collection. However, our EBC device has advantages in size, weight, and simplicity. In our study, we used a 16 cm long straw for exhaling toward to the super hydrophobic surface without any control of saliva for the possible contamination. However, our collection time was only 1–4 min, and during such short sampling period the sample contamination by saliva is very limited given the length of the straw. Another advantage of our developed device is the one time use of the hydrophobic parafilm (disposable) and exhalation straw with an easy collection of EBC, which thus prevents the possible cross contamination and facilitates the collection of EBC samples from a large number of subjects. This is particularly useful during an influenza outbreak or a man-made bio-terrorism attack in which a rapid screening of exposed persons needs to be conducted immediately.

Here, the EBC samples collected by the developed device from seven human subjects recruited from a respiratory unit of Peking University Third Hospital in Beijing were studied using culturing, DNA stain, SEM and molecular methods. In this study, the particle size distributions trends in a typical exhaled breath were also measured and are shown in Figure 4. As observed in the figure, the number concentration decreased with increasing particle diameter. For bacterial size ranges (0.65–2.2 µm), a concentration level of 329 to 25819 particles/L was observed, while for larger particles of 2.2–4 µm a concentration level of 60 to 400 particles/L was obtained. In previous studies, similar particle size distribution trend in exhaled breath was also found using the OPC, although the droplet concentrations for respective size ranges were slightly different [21], [39]. Nonetheless, due to its rapid evaporation water droplet itself or those adsorbing on bacterial particles in the exhale breath will certainly affect the results obtained here. The results from OPC indicated that particles of larger than 2.5 µm only accounted for 0.4% of the total particles exhaled. According to ICRP (1994), the total lung deposition efficiency for particles larger than 2 µm is more than 80%, while for smaller particles of less than 1 µm, the deposition efficiency is less than 40%, i.e., 60% exhaled out [44]. In addition, larger particles could stick to the straw wall. Therefore, in the exhaled breath as well as those collected into DI water droplet smaller particles would dominate.

Figure 5 shows the concentrations of culturable bacterial aerosols in EBC samples collected from seven human subjects. As shown in the figure, bacterial concentration levels ranged from 693 to 6,293 CFU/m^3^. ANOVA tests indicated that there were statistically significant differences in culturable bacterial aerosol concentrations for EBC samples collected from different subjects (p-value = .0001). In a recent study, human occupants are also identified as the significant contributors for indoor bacteria, i.e., the emission rate is about 37 million gene copies per person per hour, and a distinct indoor air signature of bacteria was demonstrated to be associated with human skin, hair, and nostrils [40]. During human breathing, the bacterial particles from environmental air are continuously inhaled, some of which, i.e., smaller ones, can be exhaled out again by the lung and reside with nostrils. Here, bacterial species *Sphingomonas* paucimobilis and *Kocuria* rosea were detected using Vitek2 in six EBC samples as shown in Table S2. Because of limitation of Vitek 2, certain bacterial species were not identified in our study. Among the subjects, subject #6 had substantially higher culturable bacterial concentrations than other subjects. From his medical conditions shown in Table S2, it was likely that his fever was caused by the bacterial infections. In his EBC sample, we found Kocuria variants which were thought to cause catheter-related bacteremia [41]. For other human subjects, the culturable bacterial aerosol concentration levels ranged from 700 to 3000 CFU/m^3^ and *Sphingomonas* paucimobilis, a non-fermenting Gram-negative bacillus, were detected. In a previous study, *S.* paucimobilis was found to cause nosocomia bacteremia outbreak [42]. For negative control samples, we did not observe the bacterial growth, indicating no contamination during the EBC collection. Ideally, bacterial particles in EBC should be collected using a suitable size-selective sampling tool to investigate the bacterial counts for different size range. However, such device is currently not available yet. Compared to the environmental culturable bioaerosol concentrations, those in EBC samples collected had relatively higher levels, thus representing an important source of bioaerosols particularly in a high human occupancy environment. In addition to viruses, *Rhodococcus equi*, a bacterium causing pyogranulomatous bronchopneumonia, were detected in the exhaled air from foals in a recent study [43]. When pathogenic bacteria are breathed out, they could pose a serious public health threat.

Figure 6 shows the qPCR amplification plot from EBC samples collected from seven human subjects in a respiratory clinic. As observed from the figure, bacterial samples were successfully amplified (Ct values were 16–19), while the positive sample (*B. subtilis*) had a Ct value of 15 and the negative control had a value of 28. Based on the DNA standards used, the concentrations of bacterial DNA in the EBC samples (Sample 1–7) were in the range of 0.32 µg/µL–3.15 µg/µL. Detection of the bacterial DNA in EBC samples was also confirmed by the melting curve of qPCR amplification as shown in Figure 7. As observed in the figure, most EBC samples had a peak at 68°C, the same as that of the positive control *B. subtilis*. For a few different peaks observed, they might be the possible primer dimer (PD) from the PCR non-specific amplification process. In addition to the qPCR amplification of bacteria in EBC samples collected, DNA stain (AO method) was also performed and the results are shown in Figure S2. As observed in the figure, both viable (green) and dead (yellow) were found in the EBC samples collected and the positive control *B. subtilis* samples, while no cells were detected in the negative control. SEM images with different resolutions and agar plate culturing shown in Figure 8 also indicated that EBC samples (cultured) had various types of bacteria based on their morphologies and colony color. From SEM images, it can be estimated that most bacteria are in the range of 0.5–1.0 µm. According to total particle deposition curve developed by ICRP (1994) [44], more than 60% of bacterial particles of below 1 µm could be exhaled out. These smaller bacterial particles could remain airborne for a prolonged time period, thus playing an important role in airborne transmission of potential diseases. Results shown in Figures 5, 6, 7, and 8 indicate that high levels of bacterial aerosols were detected in the EBC samples collected, and the results on the other hand also implied that the developed device was efficient in collecting bacterial particles in the exhaled breath. These experimental data further confirm that exhaled breath is an important source of bacterial aerosols in the built environments.

In this study, qPCR was also applied to detecting influenza A H3N2 viruses in EBC samples collected by the device. As observed in Figure S3, H3N2 viruses were detected in the EBC sample collected from subject #3 with a Ct value of 28, while those for subject #1, #2 were shown below the detection limits. In addition, spiking viruses into the samples in general enhanced the overall qPCR signal as observed in Figure S3. This on the other hand suggests no inhibition or amplification occurred when amplifying H3N2 viruses in EBC samples using qPCR. According to information shown in Table S2, subject #3 had a fever, but no other information was available at the time of the experiment. In a previous study, it was indicated that use of the RTube for EBC collection had a very low viral detection rate (7%) compared to nasal swabs (46.8%) [20]. Recently, a mask-like sampler was also tested and proved to be useful in detecting viruses using PCR in exhaled breath [23]. It was indicated that airborne virus detection is difficult due to their low concentration and the presence of a wide range of inhibitors, thus optimized molecular biology should be performed to enhance their detection [45]. Although the number of the subjects tested is limited here, the developed method, i.e., EBC collection and qPCR application, was demonstrated successful in detecting viruses from human exhaled breath. This would offer a non-invasive method for diagnosis of respiratory infections by using EBC. In the future, more patients should be tested with the EBC collection device developed here for viral detections.

Exhaled breath holds great promise for monitoring human health and for the diagnosis of various lung and systemic diseases, but analysis challenges remain due to the complex matrix of the breath [46], [47]. In this study, different from available devices restricted solely to condensation a simple and low cost EBC collection method using impaction and condensing was developed here for collecting bacteria and virus particles. An important advantage is the reusability of the collection device with a disposable hydrophobic film and an exhalation straw yet with a rapid EBC collection. This would offer the opportunity to collect EBC samples from a large number of subjects, especially during an influenza outbreak or a man-made bioterrorism event, within a shorter time frame. The developed EBC collection method was shown highly successful in detecting bacteria in EBC samples in a clinical setting. The developed EBC collection method was also shown applicable in detecting influenza viruses too. Experimental data here also suggest that exhaled breath, which was shown to contain smaller bacterial particles, could play an important role in airborne transmission of potential diseases. The collection efficiency of other substances including bio-markers (NO,CO, 8-isoprostane, hydrogen peroxide, nitrite, volatile organic compounds) using the developed method here is subject to further investigations. In addition, different exhalation modes should be also investigated with the method in collecting EBC. Besides, the dynamics of the air flow, mixing, and effects of temperatures and humidity, condensation, evaporation, growth of particles during the collection as well as the optimal straw length should be also investigated for improving the developed technique. Overall, our developed method here could be easily made available to a laboratory, and have impacts on current practice of EBC collection. Nonetheless, the reported work is a proof-of-concept demonstration, and its performance in non-invasive disease diagnosis such as bacterimia and virus infections needs to be further validated including effects of those influencing factors described.

## Supporting Information

Figure S1 **Experimental procedures used in this study include physical characterization and molecular analysis of the EBC collection efficiencies of the device and its pilot application in a respiratory clinic.**
(TIF)

Figure S2 **Optical images of EBC samples stained by Acridine Orange (AO): Bacillus subtilis species were used as the positive control and DI water was used as the negative control.**
(TIF)

Figure S3 **Detection of H3N2 influenza viruses in EBC samples collected from three human subjects with ID: 1, 2, 3 corresponding to those listed in Table S2; In addition, spiked H3N2 virus samples were also amplified; H3N2 viruses were used as the positive control and DI water was used as the negative control.**
(TIF)

Table S1 **Characteristics of widely used EBC collectors.**
(DOCX)

Table S2 **Medical conditions of seven human subjects visiting a respiratory clinic whose exhaled breath condensate samples were collected in this study.**
(DOCX)

Information S1 **PCR test and Acridine Orange stain.**
(DOC)
