# Supplementary figures and images for: Molecular and Microscopic Analysis of Bacteria and Viruses in Exhaled Breath Collected Using a Simple Impaction and Condensing Method
Source: PLoS One. 2012 Jul 25;7(7):e41137. doi: 10.1371/journal.pone.0041137 (PMC3405091; doi:10.1371/journal.pone.0041137)

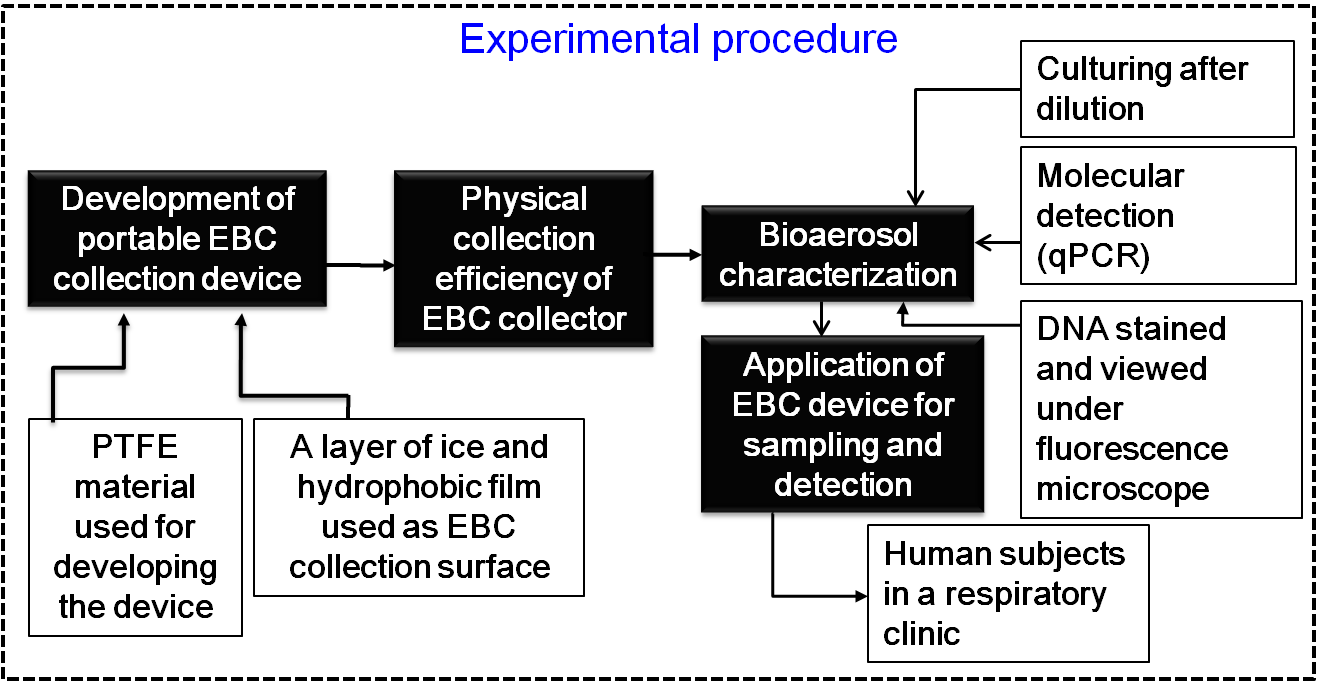

Supplement: Figure S1 — Experimental procedures used in this study include physical characterization and molecular analysis of the EBC collection efficiencies of the device and its pilot application in a respiratory clinic. (TIF) [file pone.0041137.s001.tif]

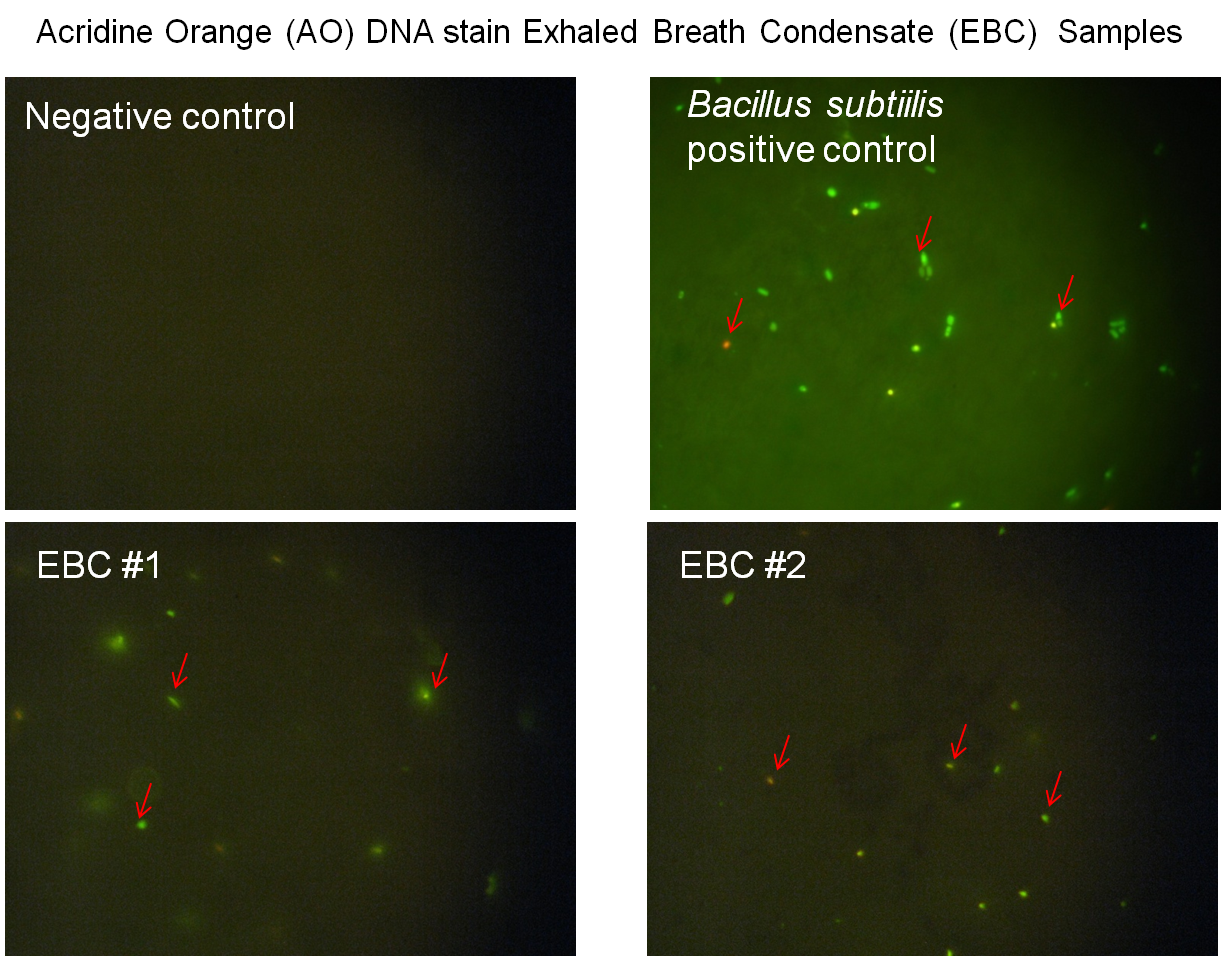

Supplement: Figure S2 — Optical images of EBC samples stained by Acridine Orange (AO): Bacillus subtilis species were used as the positive control and DI water was used as the negative control. (TIF) [file pone.0041137.s002.tif]

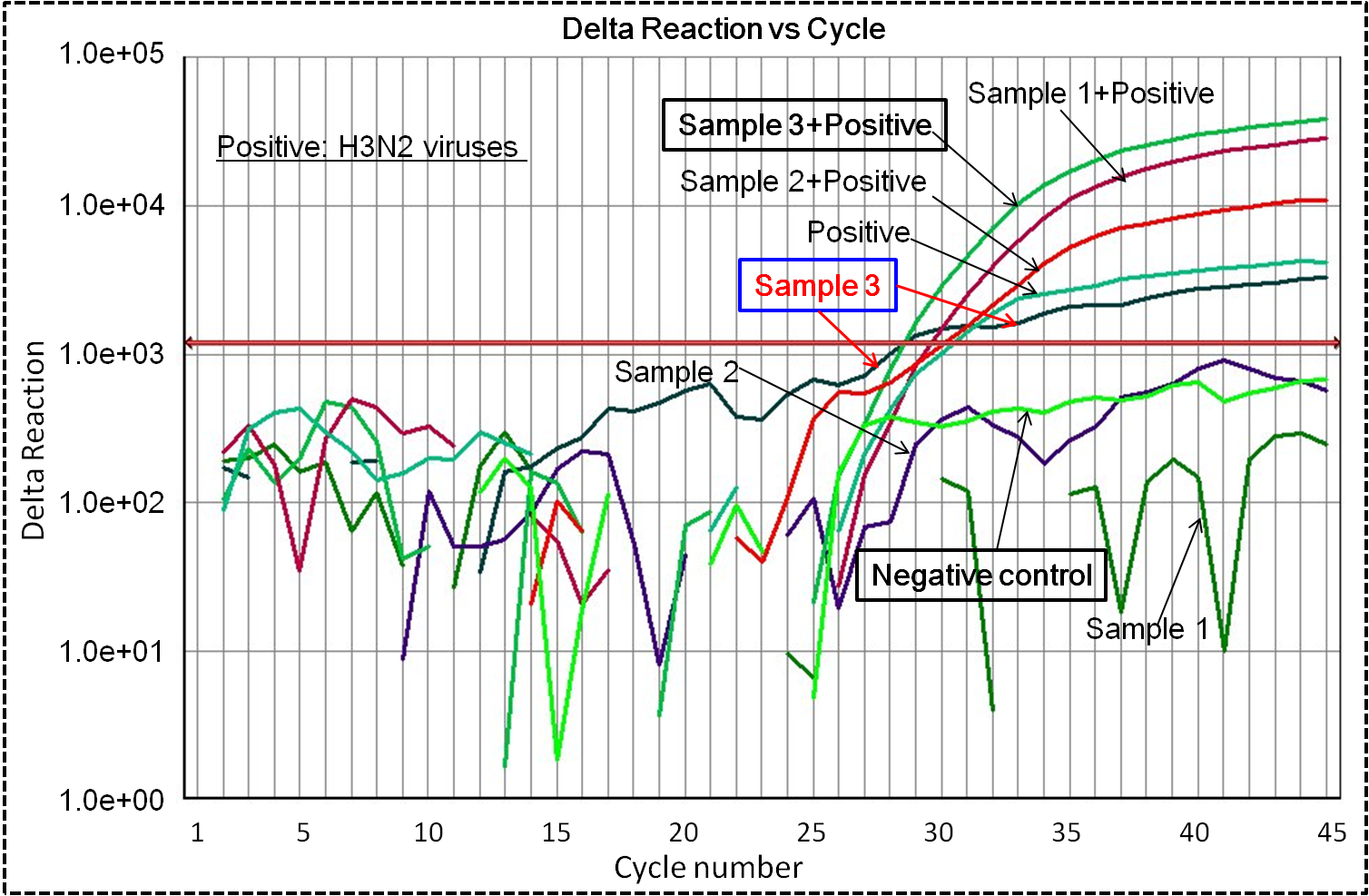

Supplement: Figure S3 — Detection of H3N2 influenza viruses in EBC samples collected from three human subjects with ID: 1, 2, 3 corresponding to those listed in Table S2; In addition, spiked H3N2 virus samples were also amplified; H3N2 viruses were used as the positive control and DI water was used as the negative control. (TIF) [file pone.0041137.s003.tif]
