# Supplementary material for: Molecular and Microscopic Analysis of Bacteria and Viruses in Exhaled Breath Collected Using a Simple Impaction and Condensing Method
Source: PLoS One. 2012 Jul 25;7(7):e41137. doi: 10.1371/journal.pone.0041137 (PMC3405091; doi:10.1371/journal.pone.0041137)
Supplement: Table S1 — Characteristics of widely used EBC collectors. (DOCX) [file pone.0041137.s004.docx]

**Table S1** Characteristics of widely used EBC collectors

| Commercial EBC  devices | Materials | Size (mm; length X width X height) | Weights | Collection temperatures | Physical collection efficiencies |
| --- | --- | --- | --- | --- | --- |
| Rtube® | Polyethylene;  O-Ring by PTFE (Teflon) | 290 X 35 X 100 | 442 g | -20°C | 1,000 μL/7 min for adult;  700 μL/10min for child (from manufacture) |
| EcoScreen® | EcoScreen® | 290X160X200 | 23 kg | -10°C | 1,880 μL/10 min (Soyer et al., 2006) |
| Anacon® |  |  |  | −20 °C | 1,350 μL/10 min (Czebe et al., 2008) |
| TurboDeccs |  | 200 X 160 310 | 5 kg |  | NA |
| Closed glass condenser system | Glass |  |  | 0°C | 465 μL/11 min (Rosias et al., 2010) |
| Our device | polytetrafluoroethylene (PTFE) material | 100 x 50 x 30 | 105 g | Film treated by -70^o^C | 100 μL/min |

**References**

Czebe K, Barta I, Antus B, Valyon M, Horváth I, et al. (2008) Influence of condensing equipment and temperature on exhaled breath condensate pH, total protein and leukotriene concentrations. Respir Med 102: 720-725.

Rosias PPR, Robroeks CM, Van De Kant KD, Rijkers GT, Zimmermann LJ, et al. (2010) Feasibility of a new method to collect exhaled breath condensate in pre-school children. Pediatr Allergy Immu 21: e235-e244.

Soyer O, Dizdar E, Keskin O, Lilly C, Kalayci O. (2006) Comparison of two methods for exhaled breath condensate collection. Allergy 61: 1016-1018.
