# Supplementary material for: Molecular and Microscopic Analysis of Bacteria and Viruses in Exhaled Breath Collected Using a Simple Impaction and Condensing Method
Source: PLoS One. 2012 Jul 25;7(7):e41137. doi: 10.1371/journal.pone.0041137 (PMC3405091; doi:10.1371/journal.pone.0041137)
Supplement: Table S2 — Medical conditions of seven human subjects visiting a respiratory clinic whose exhaled breath condensate samples were collected in this study. (DOCX) [file pone.0041137.s005.docx]

**Table S2** Medical conditions of seven human subjects visiting a respiratory clinic whose exhaled breath condensate samples were collected in this study.

| Subject ID | Gender | Age | Medical conditions | Dominant culturable bacterial species identified using Vitek 2 |
| --- | --- | --- | --- | --- |
| 1 | F | 23 | **Basic symptoms:** Fever for 4 hours, 38.8 ^o^ C (maximum temperature), cough for 1 day  **Routine blood test:** White blood cells (WBC, 7.32 x 10^9^/L , normal), red blood cells (RBC, 4.16 x 10^12^/L, Hemoglobin (HGB, 128 g/L), platelets (PLT, 257x10^9^/L), neutrophils 74.9% (⭡), lymphocytes (13.3%, ⭣), monocytes (10.7%, ⭣), eosinophils (0.7%), basophils (0.4%)  **Diagnosis:** upper respiratory tract infection | *Sphingomonas* paucimobilis  *Kocuria* rosea  Ps.stutzeri |
| 2 | M | 22 | **Basic symptoms:** Fever for three days, maximum body temperature 39^o^C, Cough for 3 days, sputum, sore throat, headache (with some improvement)  **Routine blood test:** WBC (10.9 x 10^9^/L, ⭡), HGB (127 g/L), PLT (101 x 10^9^/L), neutrophils (70.1%), lymphocytes (23.5%) | Not available |
| 3 | F | 34 | **Basic symptoms:** 38.5^o^C (maximum body temperature), Pulse rate: 105 times/min  Breathing rate: 18 times/min , Blood pressure: 110 mmHg/75 mmHg | Not available |
| 4 | M | 29 | **Basic symptoms:** Fever for 1 day, 38.5 °C(maximum temperature), cough for 1 day  **Routine blood test:** WBC (7.08x 10^9^/L, normal), HGB (159 g/L), PLT (187 x 10^9^/L), neutrophils (73.8%, ⭡), lymphocytes (16.9%, ⭣), monocytes (7.8%, normal), addicted acid cells (1.4%), basophils (0.1%) | *Kocuria* rosea  *Sphingomonas* paucimobilis |
| 5 | F | 56 | **Basic symptoms:** Fever for one day, 39.2 ^o^C (maximum body temperature), no cough, sputum, nasal congestion, runny nose, no sore throat, headache, muscle and joint pain, no other discomfort.  **Routine blood test:** WBC (7. 2 x 10^9^/L, normal), RBC (4.57 x 10^12^/L), HGB (136 g/L), PLT (159 x 10^9^/L), neutrophils (80.3%, ⭡), lymphocytes (14%, ⭣), monocytes (5%, normal), eosinophils (0.4%), basophils (0.3%) | sphmon.paucimobilis  *Bacillus* lentus  *Sphingomonas* paucimobilis  *Aerococcus* viridians  *Bacillus* firmus |
| 6 | M | 38 | **Basic symptoms:** had body temperature 38^o^ C, running rose | *Kocuria rosea*  *Kocuria kristinae* |
| 7 | M | NA | Not Available (NA) | *Staph. xylosus* |
