# Supplementary material for: Molecular and Microscopic Analysis of Bacteria and Viruses in Exhaled Breath Collected Using a Simple Impaction and Condensing Method
Source: PLoS One. 2012 Jul 25;7(7):e41137. doi: 10.1371/journal.pone.0041137 (PMC3405091; doi:10.1371/journal.pone.0041137)
Supplement: Information S1 — PCR test and Acridine Orange stain. (DOC) [file pone.0041137.s006.doc]

**Molecular and Microscopic Analysis of Bacteria and Viruses in Exhaled Breath Collected Using a Simple Impaction and Condensing Method**

Zhenqiang Xu^1^, Fangxia Shen^1^, Xiaoguang Li^2^, Yan Wu^1^, Qi Chen^1^, Xu Jie^2^, Maosheng Yao^1,*^

^1^State Key Joint Laboratory of Environmental Simulation and Pollution Control, College of Environmental Sciences and Engineering, Peking University, Beijing 100871, China

^2^ Department of Infectious Disease, Peking University Third Hospital, Peking University, Beijing 100191,China

PLoS One

*Corresponding author:

Maosheng Yao, yao@pku.edu.cn, +86 010 6276 7282

State Key Joint Laboratory for Environmental Simulation and Pollution Control, College of Environmental Sciences and Engineering, Peking University, Beijing 100871, China

Beijing, China

June 28, 2012

**Supporting Information**

**Total bacterial aerosol concentration and detection of H3N2 viruses by qPCR**

The qPCR analysis was applied to analyzing the total bacterial aerosol concentrations in the exhaled breath condensates collected from the 7 patients. For the exhaled breath collected in this study, 40 μl of each sample was taken out for DNA extraction by a bacteria DNA extraction kit (Tiangen Co., Beijing) according to the manufacturer’s instruction. The extracted DNA samples were further suspended into 50 µl DI water. Bacterial universal forward primer 5´-TCCTACGGGAGGCAGCAGT-3´(Tm, 59 ± 4 ^o^C), the reverse primer 5´-GGACTACCAGGGTATCTAATCCTGTT-3´(Tm, 58 ± 1 ^o^C) and the probe (6-FAM)-5´-CGTATTACCGCGGCTGCTGGCAC-3´-(TAMRA) (Tm, 69 ± 9 ^o^C) designed by Nadkarni and co-workers *(*Nadkarni et al., 2002*)* were used for qPCR tests. The qPCR reaction mixture (total volume was 25 μL) included 2 μL DNA template, 1 μL forward primer (10 μM), 1 μL reverse primer (10 μM), 12.5 μL 2XMaster Mix (10X Taq Buffer, dNTP Mixture, Taq (2.5 U/ μL)) (Tiangen Co., Beijing) and 12.5 μL dd H_2_O. The cycle conditions were: 50°C for 2min, 95 °C for 10 min and 40 cycles of [95 °C for 15 s and 60 °C for 1 min]. DI water (free of DNA and RNA) and *Bacillus subtilis* as DNA standards were used as the negative and positive controls, respectively, in the PCR experiments.

In addition, RT-qPCR was also applied to detecting H3N2 viruses in the EBC samples collected from human subjects #1, #2, and #3. RT-qPCR reaction mixture (total volume was 25 μL) included 5 μL DNA template, 12.5 μL RT-PCR reaction solution, 1 μL enzyme mixing solution, and 2.5 μL Influenza H1 virus reaction solution (Shuoshi Co., Jiangsu Province, China) and 2.5 μL deionized H_2_O (free of RNA enzyme). The cycle conditions were: reverse transcription reaction at 50 ^o^C for 30 min, 95 ^o^C for 5 min, 45 cycles of [95 ^o^C for 10 sec and 55 ^o^C for 40 sec]. The fluorescence signal detection of RNA samples was detected by Applied BioSystem 7300 (Life Technologies Co. Ltd. Carlsbad, California, US). DI water (free of DNA and RNA) and H3N2 RNA were used as the negative and positive controls, respectively, in the RT-qPCR experiments.

**Acridine Orange stain of EBC samples**

In this study, DNA stain of EBC sample by Acridine Orange (AO) was also conducted to further confirm the bacterial presence. 15 μL EBC sample was taken from four EBC samples collected and 15 μL AO (Glenview, IL, US) was added to each of them. The samples were then stained for 15 min in the dark. Following this step, approximately 2-3 μL stained EBC sample was photographed under fluorescence microscope Olympus CX 41 (Minneapolis, MN). DI water (free of DNA and RNA) and purified *B. subtilis* suspensions were used as the negative and positive controls, respectively. In addition, EBC samples collected from human subjects were also cultured using liquid Trypticase Soy Agar(Becton, Dickson and Company, Sparks, MD), and Scanning Electron Microscope (SEM) (S4800, Hitachi Company, Tokyo, Japan) was utilized to study the morphologies of the culturable bacteria in obtained EBC samples.

**References**

Nadkarni MA, Martin FE, Jacques NA, Hunter N (2002) Determination of bacterial load by real-time PCR using a broad-range (universal) probe and primers set. Microbiology 148: 257-266.
